# Research on the crisis propagation in the global coal trade under the Russia-Ukraine conflict

**DOI:** 10.1038/s41598-023-42643-8

**Published:** 2023-09-24

**Authors:** Hui Huang, Jingying Zhao, Haibin Liu, Shuai Ren, Meng Liu, Haiping Liu, Feng An, Yanlei Guo, Haizhong An

**Affiliations:** 1 School of Management, China University of Mining and Technology, Beijing, 100083 China; 2 School of Economics and Management, China University of Geosciences, Beijing, 100083 China; 3 School of Economics and Management, Beijing University of Chemical Technology, Beijing, 100029 China

**Keywords:** Energy security, Energy supply and demand

## Abstract

The outbreak of the 2022 Russia-Ukraine conflict exacerbated the natural gas supply shortage in European countries. European countries restarted coal-fired power plants to maintain economic and social operations. The uneven distribution of coal resources in the world makes coal international trade inevitable. The intricate trade relations between trading countries have formed a coal trade network. When a country’s coal exports are limited due to geopolitical factors, it will cause coal supply risks. The risk will spread to more countries along the trade network, eventually leading to the collapse of the trade network. This paper builds a crisis propagation model of the coal supply under the Russia-Ukraine conflict using the cascading failure model. The results showed that the Czech Republic, Ireland, Portugal, and Bulgaria become abnormal as the proportion of coal exports *β* increases. When the Russian Federation reduced its coal exports by 80% and countries maintained only 10% coal exports against crisis, 23 European countries were the worst. Iceland, Ireland, Turkey and other countries were spread by the indirect risk and became abnormal countries. The Czech Republic and Bulgaria were spread by multiple risk and became abnormal countries.

## Introduction

Coal is the most widely distributed traditional energy source worldwide. The Paris Agreement^1^, which proposed limiting global warming to 1.5 ℃, led to a decline in coal consumption demand. However, renewable energy still entails problems such as instability and high cost. In this situation, coal remains one of the vital energy sources widely used in power generation, the chemical industry and other industries. Due to the uneven distribution of coal resources, international coal trade is essential. According to the bp Statistical Review of World Energy published in 2021^2^, as of the end of 2020, European countries accounted for only 12.8% of the world’s proven coal reserves. Coal resources endowment of European countries is low, coal resources rely more on Russian Federation. According to the U.N. Comtrade database, the number of European countries importing coal from the Russian Federation in 2022 accounted for 75.67% of the total number of countries. Among them, Turkey, Germany, Italy and other countries are the European countries that import the largest amount of coal from the Russian Federation. The intricate coal trade relations between countries have formed a coal trade network.

According to the global energy crisis analysis by the International Energy Agency^3^, in 2021, economies worldwide experienced a rapid rebound, leading to a surge in natural gas prices in Europe. Additionally, as the weather grew colder, the demand for energy, including natural gas, soared in European countries, surpassing the available supply and causing shortages. The situation was further exacerbated in 2022 by the escalation of the Russia-Ukraine conflict, intensifying the natural gas crisis in Europe. Therefore, Germany, Austria, France, the Netherlands and other countries have decided to restart coal-fired power plants^4^. However, the Russian Federation’s coal exports are affected by geopolitical wars, coal supply is limited. Geopolitics and regional resource endowment are important factors influencing international trade. In the international trade network, supply risks propagate hierarchically^5^, and commodity-intensive international trade networks are more susceptible to severe supply risk shocks^6^. In the global coal trade network, coal supply risks will gradually spread along network. In the process of propagation, when the country’s coal prices rise or coal demand is insufficient, resulting in the imbalance between supply and demand, which in turn leads to a decline in the volume of foreign trade in coal, making the related countries suffer a greater crisis. When the crisis accumulates to a certain extent, there will be systemic collapse, which is called cascading failure in complex networks. Lee et al.^7^stated that a supply crisis could be transmitted in an indirect and complex way in addition to a direct way. Specifically, even countries that do not have direct trade with countries propagating supply risks can still be affected by such risks.

Scholars often use complex network methods to build trade networks, and then recognize the structure of trade networks and identify core countries by analyzing trade network indicators and trade group structures. The study of complex networks initially focused on the characteristics of networks. In 1998, Watts and Strogatz^8^ proposed the small-world network, and in 1999, Barabasi and Albert^9^ introduced the concept of scale-free networks, which sparked a surge of research interest in complex networks. In 2003, Serrano and Boguna^10^ was the first to apply complex network theory to international trade research, revealing that international trade networks exhibit complex network features, such as scale-free distribution, small-world properties, and high clustering coefficient. Garlaschelli and Loffredo^11^ further refined the analysis of international trade networks by incorporating trade directions and temporal evolution to better capture their topological structures. In 2009, they extended their research by introducing weights and directions to construct directed weighted networks, enabling the analysis of the topological properties of the global trade network in the year 2000^12^. During this phase, scholars primarily focused on studying and analyzing the overall characteristics of international trade networks.

As research on international trade networks continues to deepen, scholars have started to investigate specific product-based international trade networks, such as energy minerals^13–16^, agricultural products^17,18^, and more. They often explore the network’s topological structure and evolutionary patterns over several years through measures such as degree, betweenness centrality, and trade communities. In the context of coal trade network construction, some scholars have built fossil energy trade networks that include coal, while others have focused solely on constructing coal trade networks to study their evolution. Gao, Sun and Shen^19^ constructed a multi-layer fossil energy trade network covering the period from 2002 to 2013 and analyzed trade communities and the time-varying evolution of main countries’ importance. Hao, An, Qi and Gao^20^ converted the weight of fossil fuels into exergy to create a directed weighted fossil energy trade network, where countries were nodes, and exergy flow values were represented as edge weights. They analyzed the overall network structure, major countries, and trade communities from1996 to 2012. Zhong, An, Fang, Gao and Dong^21^ converted the three major fossil energy sources into emergy value and built a fossil energy trade network from 2000 to 2013, analyzing the network's topological structure. Wang, Li and Cheng^22^ explored the evolution of the global coal trade network from 1996 to 2015 by using the directed weighted complex network method. Chen, Tan and Li^23^ constructed a coal trade network from 1999 to 2018, analyzing trade relationships, scale and distribution to identify core trading countries and trade hub countries.

Currently, the research models of supply crisis propagation in trade networks mainly include the epidemic, bootstrap percolation and cascading failure models. Using the epidemic model, Hao and An^24^ analysed the supply crisis propagation mechanism in the international steel trade multilayer network under the scenario of supply shortage and oversupply. However, some scholars suggest that the epidemic model does not apply to crisis propagation in economic systems. The use of the epidemic model without criticism will ignore the crisis factor analysis of the economic system. As a macroeconomic system, the international trade network has characteristics that should be included in the crisis propagation model^25,26^. In terms of the bootstrap percolation model, Chen, An, An, Guan and Hao^27^, Tian, et al.^28^ and Zhou, Cheng, You and Zheng^29^ used the bootstrap percolation model to simulate the supply crisis of natural gas, critical resources and log trade networks, respectively, and identified the core countries in crisis propagation. In terms of the cascading failure model, Sun, Shi and Hao^5^, Wang, Li, Yao, Zhu and Liu^30^, and Ren et al.^31^ used the cascading failure model to simulate the avalanche scale and avalanche propagation time of graphite, cobalt and mineral product trade networks. The core countries in crisis transmission were identified, and the supply crisis transmission process caused by the core countries was analysed. Cui, Kang and Tang^32^ used a cascading failure model to identify abnormal countries under the natural gas supply crisis caused by the Russia-Ukraine conflict and further evaluated the natural gas supply crisis. All these studies analysed the propagation time, process, and characteristics of supply crises from the cascade perspective. Given the differences between coal and other resources in terms of geographical location, supply and demand centres and trade relations, the spread of the coal trade supply crisis has its own characteristics. However, the global coal trade is also a part of international trade, and its crisis propagation is a dynamic cascade process. Therefore, it is reasonable to use a cascading failure model to simulate the supply crisis propagation of the coal trade.

Through a review of the literature on coal trade networks and supply risk, it was found that the latest available coal trade network data is from 2018. Furthermore, it was observed that although many scholars have used the cascading failure model to simulate the propagation of supply risk in trade networks, most of them relied on the original cascading failure model, which heavily depends on two parameters *α* and *β* and cannot objectively reflect real-world situations. In light of these limitations, this study makes two main contributions. Firstly, we constructed a coal trade network in 2021 and analysed the characteristics of the coal trade network in the new era. Secondly, we improved the cascading failure model by incorporating the relationship between coal supply and demand as the condition to determine when a country transitions from a normal to an abnormal state. Additionally, the *α* value was combined with the actual percentage reduction in coal exports to closely approximate real-world scenarios.

The crisis propagation model of the coal supply under the Russia-Ukraine conflict is constructed to explore the abnormal countries at low, medium, high and extreme levels. The abnormal borderlines of four European countries are obtained. And it is determined that when the Russian Federation reduces its coal exports to some countries by 80%, and stops its coal exports to other countries, only 10% of the coal exports will be retained by the countries suffering from the risk. This paper analyses the spread of coal supply risks, considering direct spread, indirect spread and multiple spread.

### Data source and processing

The national coal production data come from the 71st edition of the bp Statistical Review of World Energy published in 2022^33^ and the quarterly coal production data of the International Energy Agency^34^. The coal trade volume data are from the U.N. Comtrade database, which contains all the export and import flows (unit: Kg). The commodity name is Coal, and its H.S. code is 2701.

Since different statistical methods are used in the U.N. Comtrade database, the reported import and export volumes of the same country sometimes differ. When data are available for both the import and export countries regarding the same trade, import country data are preferred. The original data are retained when data are available for only the import or the export country regarding the same trade^5,35^. This paper also removes missing data and invalid data. To prevent observations with small trade volume from affecting the validity of the results, this paper removed observations with a trade volume of less than 10,000 Kg^36,37^. On this basis, the coal trade volume of 54 countries accounts for 82.9% of the global coal trade volume (excluding trade volumes less than 10,000 kg). This can be more representative in reflecting the overall pattern of the global coal trade^38^.

## Model construction

### Construction of the global coal trade network

The paper defines the coal trade network as a directed weighted network *G*.

1 $$\begin{array}{c}G=\left(V,A,E,W\right),\end{array}$$

2 $$\begin{array}{c}V=\left({v}_{i}, i=\mathrm{1,2},\dots ,n\right),\end{array}$$

3 $$\begin{array}{c}E=\left({e}_{ij}, i,j=\mathrm{1,2},\dots ,n\right),\end{array}$$

where *V* represents the country participating in the global coal trade. The number of countries is *n*. *A* shows the node’s attribute, representing the coal production of the countries participating in the global coal trade. *E* is the trade link of the countries. *W* represents the edge weight. When the value of *e*_*ij*_ is 0, the value of *w*_*ij*_ is also 0.

### Crisis propagation model of the coal supply

This paper uses a cascading failure model to simulate the impact of the Russia-Ukraine conflict on the global coal trade network. The specific steps to build the model are as follows.

#### Define the initial load and capacity

The initial load of country i is *O*_i_, which is the coal production of country i. The capacity of country i is *C*_i_, which is the coal consumption of country i. The formula is as follows:

4 $$\begin{array}{c}{C}_{i}=\sum_{h=1}^{{m}_{1}}{e}_{hi}+{O}_{i}-\sum_{l=1}^{{m}_{2}}{e}_{il},\end{array}$$

where *C*_i_ represents the capacity of country i, *e*_*hi*_ is the coal import volume from country i to country h, *m*_1_ is the number of countries exporting coal from country i, *e*_*il*_ is the coal export volume from country i to country l, *m*_2_ is the number of countries importing coal from country i, and *O*_i_ represents the coal production of country i.

#### Define the state of the trading country

In the trade network, countries have two main states, normal and abnormal. The normal state includes countries in the trade network whose coal trade volume is not affected and countries whose coal trade volume is affected but whose coal supply is not lower than the coal demand. The abnormal state refers to an apparent shortage of coal capacity of trading countries in the trade network, in which the coal supply is lower than the coal demand. In the initial network, all countries are in a normal state.

#### Define assumptions

The paper makes the following assumptions by collecting news media reports and combining them with the actual situation. ① The Russian Federation stops exporting coal to the EU, Switzerland, Britain and Ukraine^39^. ② The EU stops exporting Russian Federation coal. ③ Ukraine stops exporting coal to all countries^40^. ④ Governments do not intervene in crisis propagation immediately. During the model's operational period, countries that have already become abnormal states will not transition back to a normal state. ⑤ Inventory is not considered in the coal consumption of each country. The coal production and the coal obtained through trade by each country constitute the coal consumption for this year. ⑥ The structure of the global coal trade network does not change. The occurrence of coal supply risk does not lead to the emergence of new trade relationships.

#### Define conditions of the abnormal state

When regional disputes arise or countries’ relationships deteriorate, trade country *v*_r_ (the Russian Federation) stops or reduces coal trade cooperation with other trade countries *v*_i_ and *v*_j_ (the list of unfriendly countries published by the Russian Federation). This paper uses *v*_r_ as the source trade country and *v*_i_ and *v*_j_ as the target trade countries. The source trading country *v*_r_ thus changes from a normal state to an abnormal state. In combination with reality, the cessation of coal trade cooperation shows that the export volume of the source trading country *v*_r_ to some target trade countries *v*_i_ (including the EU, Switzerland, Britain and Ukraine) decreases to 0. Moreover, the reduction in coal trade cooperation shows that the export volume of source trade country *v*_r_ to some target trade countries *v*_j_ decreases by the fraction *α*. We set α 20%, 50%, 80% and 100%, representing low, medium, high and extreme levels respectively^28^. The formula is as follows:

5 $$\begin{array}{c}{e}_{ri}{\prime}=0,\end{array}$$

6 $$\begin{array}{c}{e}_{rj}{\prime}=\left(1-\alpha \right){e}_{rj}\left(0\le \alpha \le 1\right),\end{array}$$

where *α* represents the proportion of the export reduction of the source trade country to each target trade country in the export volume. *e*_*ri*_*′ *represents the coal export volume from country r to country i after the Russia-Ukraine conflict. *e*_*rj*_*′*shows the coal export volume from country r to country j after the Russia-Ukraine conflict. $e_{rj}$ is the coal export volume from country r to country j in the initial global coal trade network.

It is understood that coal shortages are the leading cause of the coal crisis, and coal shortages cause an imbalance between the supply and demand of coal, which has a harmful impact on economic and social development^41^. This paper defines the abnormal state as follows. When the coal supply of the target trade countries *v*_i_ and *v*_j_ is lower than the coal demand of these countries, the target trade countries *v*_i_ and *v*_j_ change from a normal state to an abnormal state. At this time, *v*_i_ and *v*_j_ change from the last edge's target trade country to the current edge’s source trade country. The countries that import coal from source trade countries *v*_i_ and *v*_j_ then change to the target trade country of the current edge. Considering that in order to resist crisis, countries reduce the proportion of coal exports to the fraction *β*. In order to explore the impact of the Russia-Ukraine conflict on European countries at four levels, and considering that the country still suffers from the risk after reducing its export proportion, the article assumes that after the country becomes the source trade country, the export volume of source trade country to target trade countries decreases by the fraction *α*, when a country fails to resist crisis after reducing exports and becomes a source trade country. The coal demand of each country is represented by the coal consumption. The formula is as follows:

7 $$\begin{array}{c}\sum_{h=1}^{{m}_{1}}{e}_{hi}{\prime}+{O}_{i}-\beta \sum_{k=1}^{{m}_{2}}{e}_{ik}{\prime}<{C}_{i},\end{array}$$

where *e*_*hi*_*′ *refers to the coal export volume of country h to country i after the Russia-Ukraine conflict. *m*_1_ is the number of countries importing coal from country i. *e*_*ik*_*′*represents the coal export volume from country i to country k. *m*_2_ shows the number of countries exporting coal from country i. *ß* is the coal export proportion, which is the proportion of the export volume after the Russia-Ukraine conflict to the export volume before the Russia-Ukraine conflict.

#### Run the model

**Figure 1:**
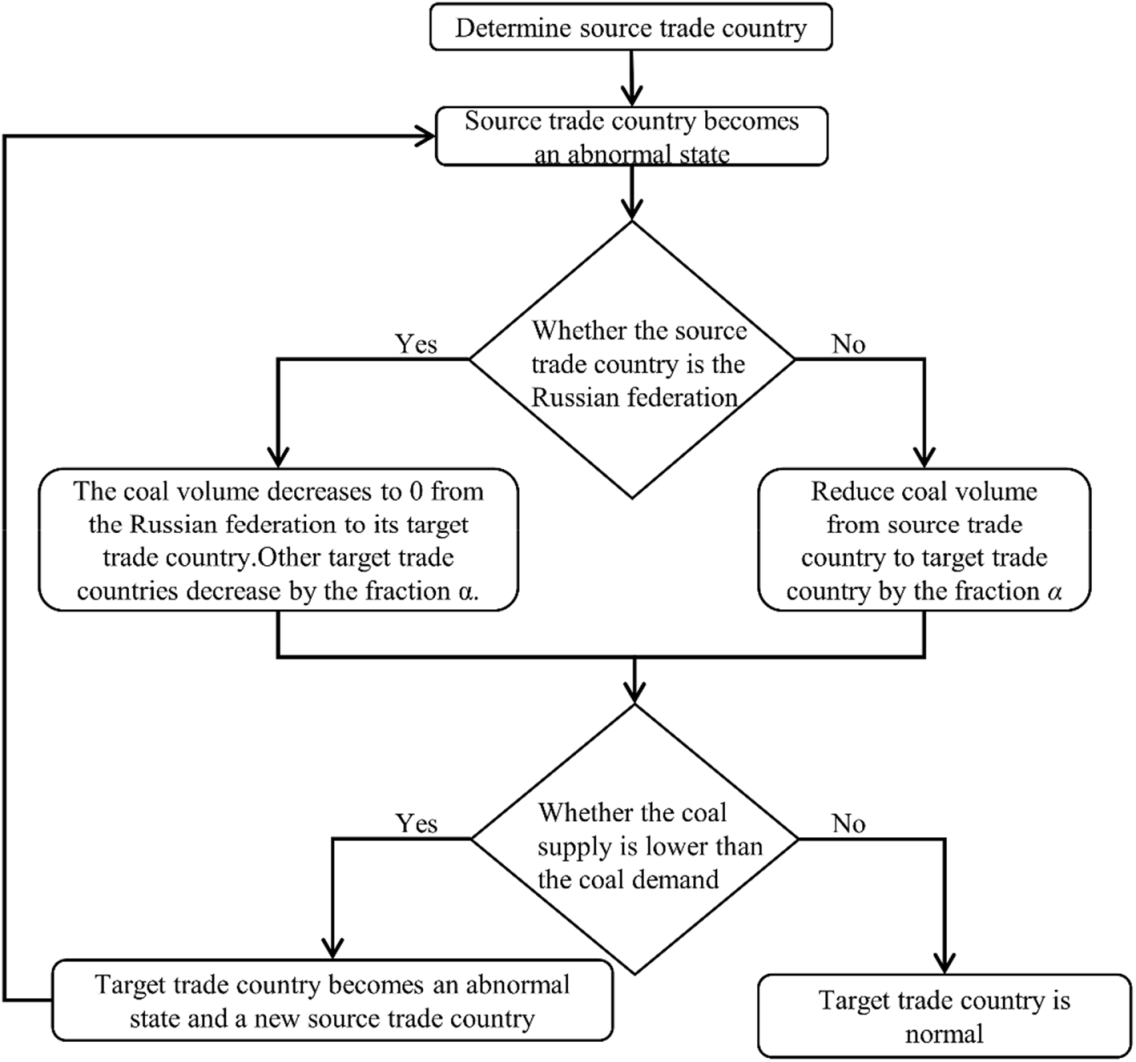
Cascading failure model process.

This paper simulates the impact of the Russia-Ukraine conflict on the global coal trade network until there are no new countries in an abnormal state in the network. Figure 1 shows the process of the cascading failure model.

## Crisis propagation of the coal supply under the Russia-Ukraine conflict

The impact of the Russia-Ukraine conflict on the global coal trade is simulated by building a cascading failure model. The Russian Federation will reduce coal exports to unfriendly countries to Russia, or even completely stop coal exports. The above countries are divided into two categories, as shown in Table 1. The article divides *α* into four levels, including low, medium, high and extreme levels, which are 20%, 50%, 80% and 100% respectively. The following is an analysis of the abnormal state of European countries and the crisis propagation of coal supply under the four levels.

**Table 1:** The type of countries.

Country	Name
Romania, Denmark, Slovakia, Czech Republic, Hungary, Austria, Belgium, Italy, Sweden, Netherlands, France, Poland, Germany, Luxembourg, Greece, Spain, Slovenia, Latvia, Estonia, Lithuania, United Kingdom, Ukraine, Switzerland	Type A countries
Australia, USA, Japan, Norway, Canada	Type B countries

**Figure 2:**
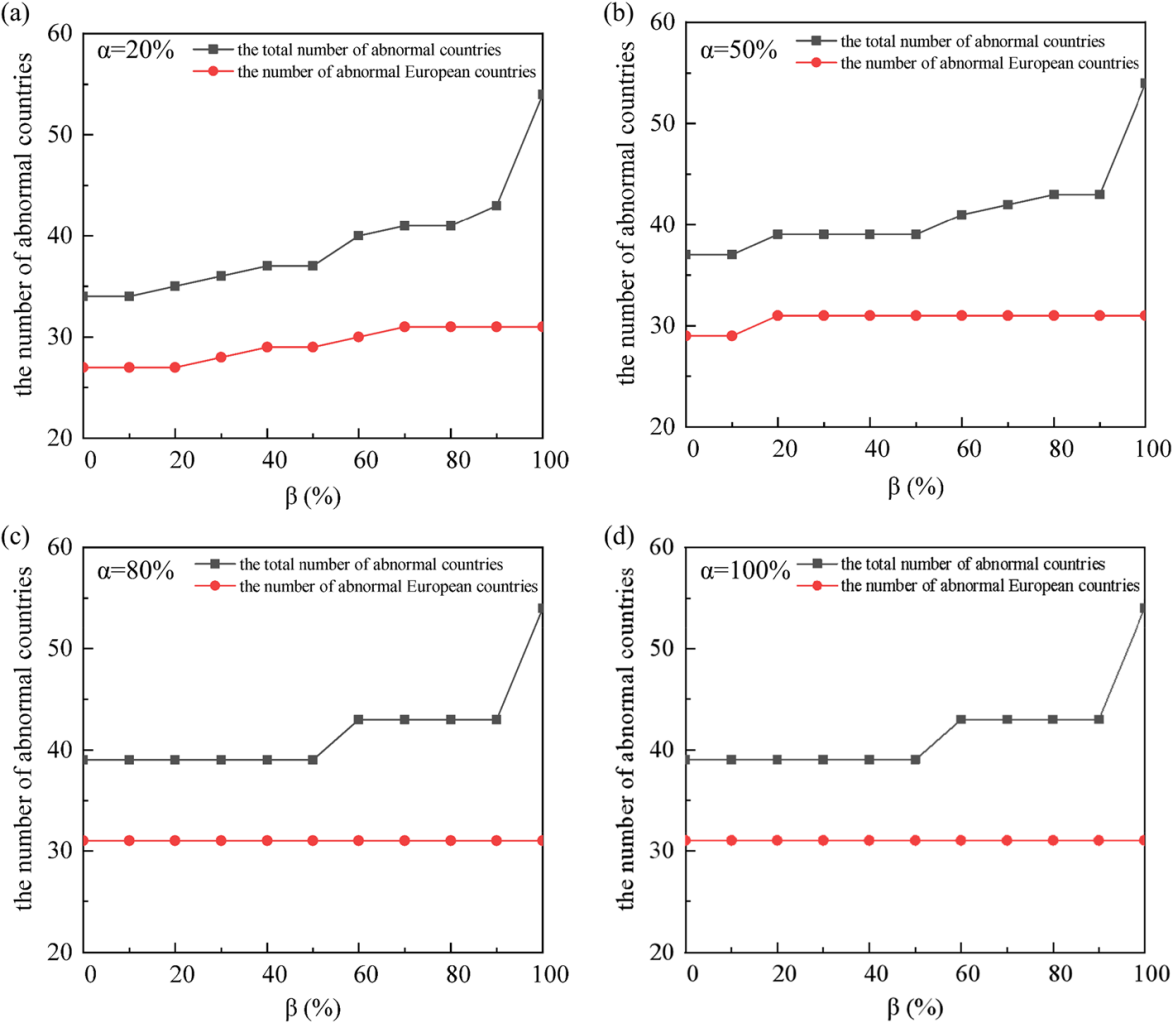
The number of abnormal countries at low, medium, high and extreme levels (note: there are 31 European countries except the Russian Federation).

Figure 2 shows the changes in the number of abnormal countries at the low, medium, high and extreme levels. Through simulation, it is found that when the Russian Federation stops exporting to type A countries and reduces exports to type B countries by 20%, and type A and type B countries do not reduce their coal exports, the whole network collapses and all European countries suffer risks. However, when the countries reduce coal exports to ensure domestic coal supply, the total number of abnormal countries decreased significantly. When the coal export proportion is [0%, 20%], 27 European countries become abnormal countries. As the proportion of coal exports *β* increases, the Czech Republic, Ireland, Portugal, and Bulgaria become abnormal.

In order to explore the crisis propagation of the coal supply among all European countries in the trade network, it is necessary to determine the shock degree α. It can be seen from the previous section that when the shock degree *α* is 20% and 50%, the European countries in the trade network do not all become abnormal. When the shock degree *α* is 100%, the abnormal countries at extreme level are unrepresentative. Therefore, we determine the shock degree *α* is 80%. At this time, no matter how the coal export proportion of the target trade country changes, all European countries in the trade network are exposed to crisis, so we determine the coal export proportion* β* is 10%.

**Figure 3:**
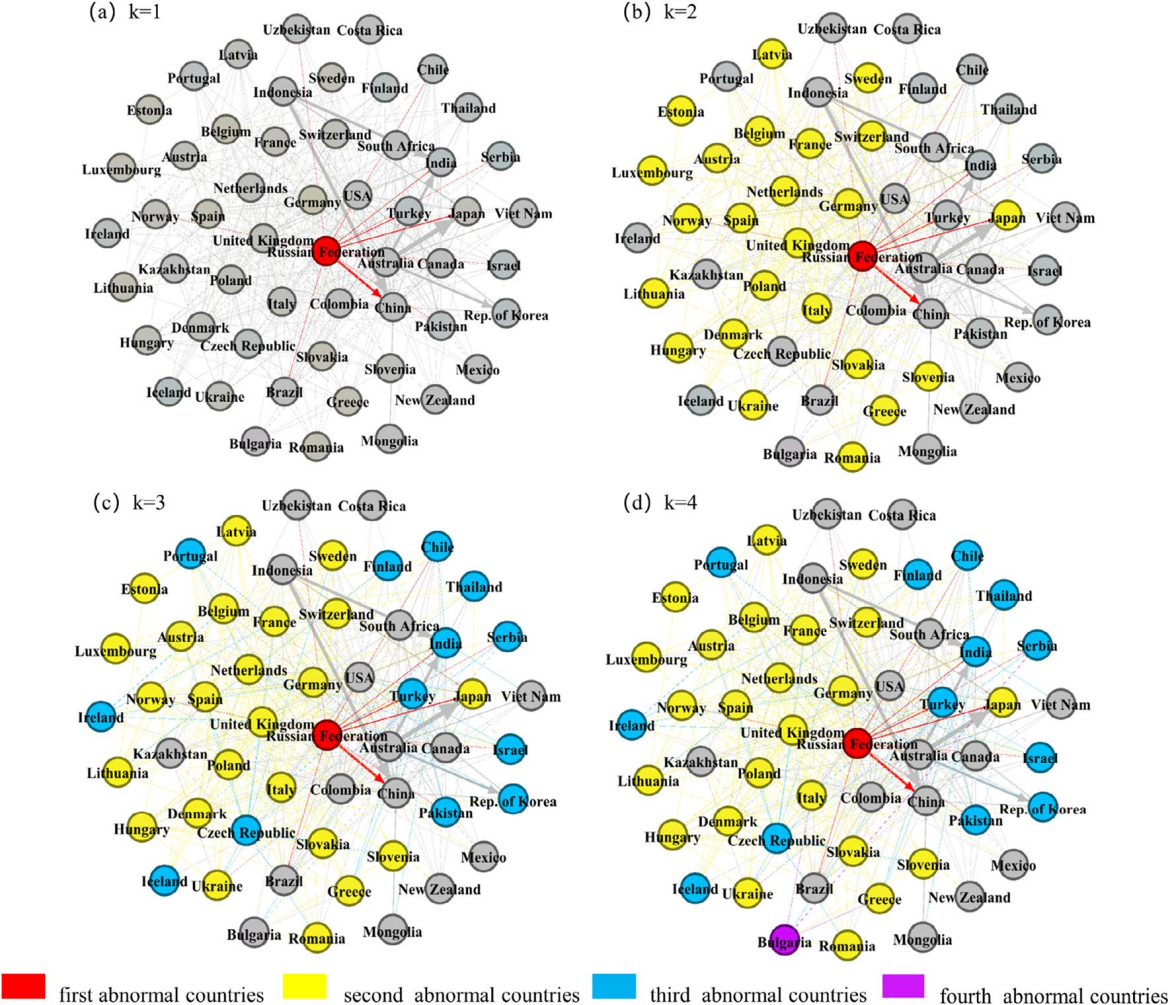
The process of crisis propagation.

Figure 3 shows the situation of countries in an abnormal state after each crisis propagation in the network, and k represents the level of crisis spread. In the first level of crisis propagation (Fig. 3a), the Russian Federation is designated as a country in an abnormal state according to the specific meaning of the shock, and then the Russian Federation imposes the next shock on the countries on its unfriendly countries list.

### Direct propagation

After the second level of crisis spread in Fig. 3b, after the Russian Federation stopped supplying coal to type A countries and reduced the coal supply to type B countries by 80%, all countries in an abnormal state, except Japan, were in Europe. This shows that Europe is at the centre of the storm in the coal supply crisis caused by the Russia-Ukraine conflict. Romania, Denmark, Slovakia, and other countries were in an abnormal state. These are 23 European countries, 19 of which belong to the European Union, and Ukraine is a part of the Russia-Ukraine conflict. On June 7, 2022, the President of Ukraine proposed stopping coal exports to ensure its coal supply^40^. However, in the simulation, Ukraine is set to cut off the supply of coal for export, and it still falls into an abnormal state in the coal supply crisis shock. Coal is Ukraine’s leading energy source, accounting for approximately 70% of primary energy consumption. On September 27, 2022, Donetsk, Ukraine’s leading coal-producing region, joined the Russian Federation through referendum^42^. Ukraine must therefore increase its coal imports to meet domestic demand. The United States, Colombia, Australia and Kazakhstan are the countries that Ukraine can rely on for imports, but the Russian Federation previously accounted for 66.87% of the total import volumes, according to the U.N. Comtrade database. Nuclear power accounted for 20% of Ukraine's electricity. However, due to the conflict, Zaporozhye, the largest nuclear power plant in Ukraine, stopped producing electricity^43^. The domestic demand for coal in Ukraine increased.

With the worsening of the Russia-Ukraine conflict, the amount of natural gas delivered by the Russian Federation to European countries has decreased significantly. On September 2, 2022, the Russian Federation announced that the “Nord Stream 1” natural gas pipeline was forced to stop operation. Following the closure of the pipeline and the lack of natural gas reserves, European countries could ease their energy crisis by reusing coal. In the past, Belgium, Austria and Sweden have stopped using coal for power generation due to strong public awareness and low coal production. Countries such as the U.K. and Germany planned to stop using coal for power generation, especially after 2018, when Germany no longer produced hard coal. However, the chain reaction triggered by the Russia-Ukraine conflict made European countries gradually realize the importance of energy security. The energy supply must be higher than the energy demand. Poland's coal power accounts for approximately 70% of its energy use, the highest proportion among EU members. Poland banned the import of coal from the Russian Federation in April 2022. The shortage of the coal supply and the soaring price of coal followed consecutively. In July 2022, the Polish Prime Minister acknowledged that Poland would face the problem of coal shortages in the near future. In this case, the Ministry of Climate and Environment of Poland cancelled the requirements for the quality of coal for heating^44^. Austria, France, the Netherlands, Spain, and other countries had to reopen coal-fired power plants. The Ruhr mining area, which produced hard coal in western Germany, also resumed operation. The United Kingdom even began to consider new coal-fired power plants^45^. Most European countries have low coal reserves and high dependence on coal, and most of them still rely on the Russian Federation^46^. When the Russian Federation cuts off the coal supply to these countries, they can easily fall into an abnormal state. In addition to the reduction in coal exports from the Russian Federation, which led to the shortage of coal in European countries, hot weather further increased the electricity demand. In June 2022, the average temperature in Spain, France, Italy and other countries was more than 10 ℃ higher than that in previous years^47^. High temperatures drive the demand for electricity and further increase the need for coal.

### Indirect propagation

**Figure 4:**
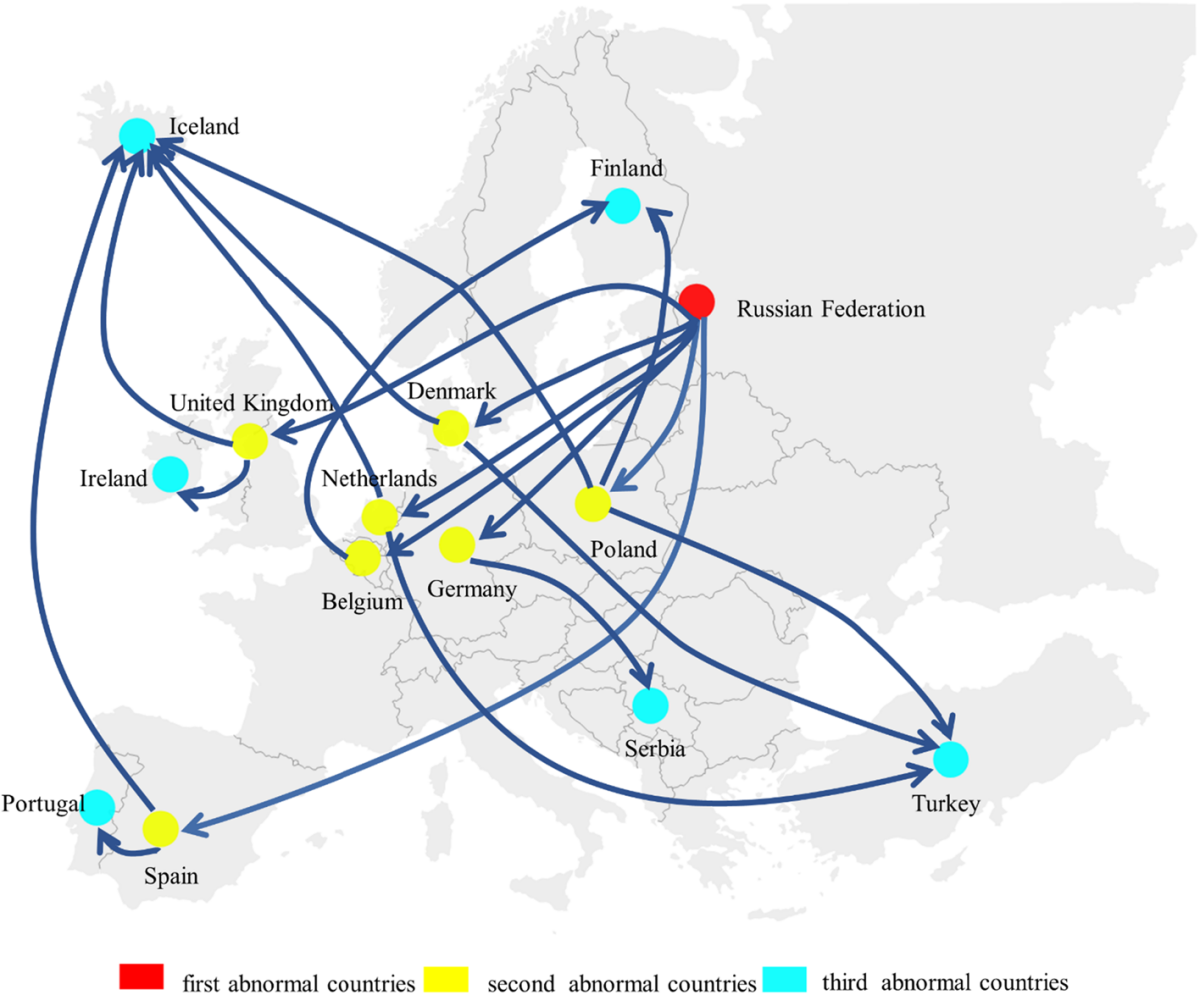
Indirect propagation.

After the third level of crisis propagation in Fig. 3c, after the Russian Federation stopped supplying coal to type A countries and reduced the coal supply to type B countries by 80%, these countries reduced the coal supply to their exporting countries by 80%, there are 13 countries in an abnormal state, seven of which are in Europe, namely, the Czech Republic, Iceland, Ireland, Turkey, Finland, Serbia, and Portugal. In the case of this indirect impact, a country can have no direct contact with the source trade country but still fall into an abnormal state. The specific propagation path is obtained after several simulations, as shown in Fig. 4. Iceland fell into an abnormal state after exposure to the coal supply crisis spread by the United Kingdom, Denmark, the Netherlands, Poland or Spain. Iceland is the second largest island country in Europe. It is a volcanic island with abundant hydropower and geothermal resources. In terms of power supply, almost 100% of its energy is renewable energy, 75% of which comes from hydropower and 25% from geothermal power^48^. Iceland’s aluminum and steel smelting industries have developed well. In these industries, coke is used as a reducing agent. Since Iceland has no coal resources, it needs to obtain coke through international trade. Ireland faced an abnormal state after being exposed to the coal supply crisis spread by the United Kingdom. Ireland is the country with the lowest dependence on the Russian Federation among EU members, but it is also among the countries with the highest dependence on imported energy in Europe^49^. It has an indirect dependence on the Russian Federation. Ireland does not directly import natural gas from the Russian Federation but from the United Kingdom^50^. However, the Russia-Ukraine conflict led to a natural gas supply shortage. Ireland had no natural gas reserves and no liquefied natural gas import facilities along the coast. Therefore, Ireland had to import natural gas only from the U.K. but the British Gas Company claimed that the natural gas inventory was sufficient for only 9 days^51^. The demand for coal in both countries greatly increased. Ireland’s coal was also mainly imported from the United Kingdom. According to the U.N. Comtrade database, Ireland’s coal import volumes from the United Kingdom accounted for 64.78% of the total import volumes in 2021. Turkey faced an abnormal state after exposure to the coal supply crisis spread by the Netherlands, Denmark or Poland. After being exposed to the coal supply crisis spread by Belgium, Germany or Poland, Finland faced an abnormal state. Finland is located in northern Europe and is one of the EU members. After the Russia-Ukraine conflict broke out, Finland tried to join the North Atlantic Treaty Organization. To prevent this, Russia cut off the natural gas and electricity supply to Finland^52^, which had a significant impact. Finland’s demand for coal increased. Poland, Belgium and Germany are the source countries of Finland’s European coal imports. According to the U.N. Comtrade database, Finland’s coal import volumes from these countries accounted for 99.55% of the total coal import volumes from all European countries in 2021. Serbia faced an abnormal state after being exposed to the coal supply crisis spread by Germany. After the Russia-Ukraine conflict, EU countries announced several rounds of sanctions against Russia, but Serbia always maintained a neutral attitude. Serbian President Vucic proposed that Serbia would not impose sanctions on the Russian Federation unless the country's survival was threatened^53^. The Russian Federation has not stopped supplying energy to Serbia under friendly trading relationships. However, Serbia is surrounded by Bulgaria, Macedonia, Hungary, Romania and other EU members and was forced to suspend its energy imports due to the impact of EU sanctions against the Russian Federation. German Prime Minister Scholtz asked Serbia to join the EU in sanctioning the Russian Federation and promised to provide help in the energy field. However, Serbia often holds different positions than the EU. Portugal fell into an abnormal state after being exposed to the coal supply crisis spread by Spain. Spain is the second largest coal import source for Portugal. According to the U.N. Comtrade database, Portugal imported 40.26% of its total coal volume from Spain in 2021.

### Multiple propagation

**Figure 5:**
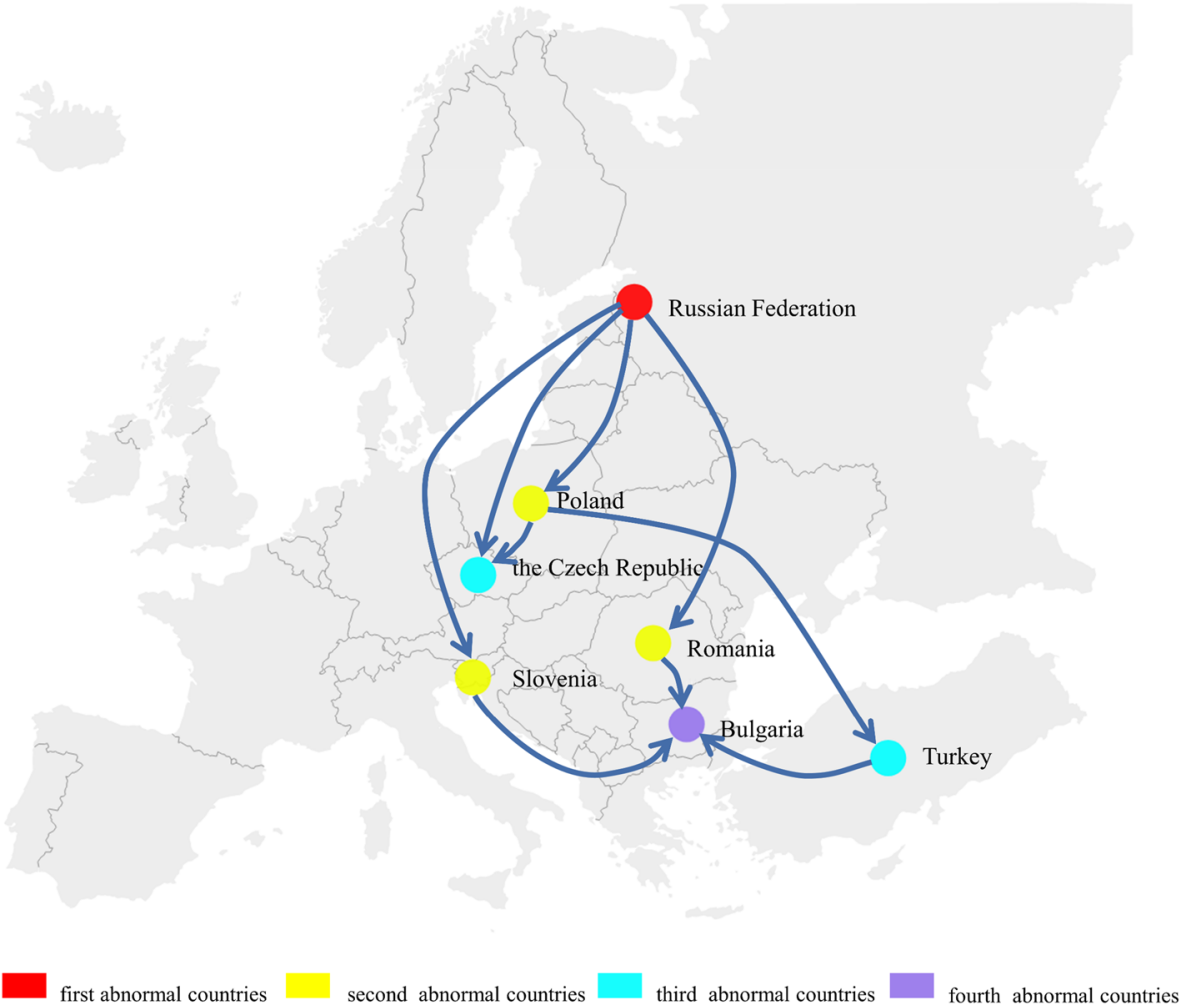
Multiple propagation.

Multiple propagation means that a country has a direct coal trade relationship with the Russian Federation, but the direct risk spread by the Russian Federation is not enough to make this country become an abnormal country. After the risk spread by other countries, this country becomes an abnormal country. In Fig. 3c, the Czech Republic fell into an abnormal state after the third level of crisis propagation. In Fig. 3d, Bulgaria fell into an abnormal state after the fourth level of crisis propagation. They are both multiple propagation of the coal supply crisis. The multiple propagation process is shown in Fig. 5. As members of the European Union, the Czech Republic and Poland faced a shock under the coal supply crisis caused by the Russia-Ukraine conflict. Poland is the largest coal-producing country in Europe and borders the northern part of the Czech Republic. According to the U.N. Comtrade database, the Czech Republic imported 82.44% of its coal resources from Poland in 2021. Therefore, Poland caused a crisis shock to the Czech Republic’s coal supply. After the Russia-Ukraine conflict began, the Polish Prime Minister admitted that there would be a shortage of coal reserves^44^. Recently, Polish residents have queued up to buy coal in the Czech Republic. This further led to the multiple coal supply risks, making the Czech Republic become an abnormal country.

Bulgaria fell into an abnormal state after the fourth level of crisis spread. In addition to the shock from Romania and Slovenia, which fell into an abnormal state after the second level of crisis spread, Bulgaria faced a shock from Turkey, which fell into an abnormal state after the third level of crisis spread. After the Russia-Ukraine conflict broke out, Bulgaria refused to pay for natural gas from the Russian Federation in rubles and transported equipment to Ukraine. No matter the sanctions imposed by EU countries on the Russian Federation, or the anti-sanctions imposed by the Russian Federation on EU countries, Bulgaria will suffer from the risk of coal supply and face the problem of domestic coal shortage. With Bulgarian President Rumen Radev appointing Galab Donev as caretaker Prime Minister, Bulgaria's attitude towards Russia has become more neutral. On October 5, 2022, according to news media reports, Bulgaria suspended the implementation of EU sanctions on Russian fuel in order to ensure the work of government agencies and ease the various sanctions imposed by the Russian Federation, in an attempt to reduce the impact of coal supply risks in this way^54^.

## Conclusions and future outlook

### Conclusion

This paper built a global coal trade network using coal trade data from 2021, analysed the coal trade network indicators and simulated the coal supply crisis propagation under the Russia-Ukraine conflict. It analysed the abnormal state of European countries and the crisis propagation of coal supply when the shock degree *α* is 80% and the export proportion *β* is 10%. Four conclusions can be drawn.

1. At a low level, after the Russian Federation stopped its coal exports to type A countries and reduced its coal exports to type B countries by 20%, 27 European countries were in an abnormal state at first. With the increase of coal export proportion, the Czech Republic, Ireland, Portugal and Bulgaria were in an abnormal state. So far, all European countries have suffered risks. At high and extreme levels, European countries failed to resist risks by taking measures to reduce coal exports to ensure domestic coal supply, and all European countries suffered risks.
2. After the Russian Federation stopped supplying coal to type A countries and reduced the coal supply to type B countries by 80%, twenty-three European countries fell into an abnormal state under the direct coal supply crisis. Europe was at the centre of this storm. On June 24, 2022, the thermal coal price in ports of Amsterdam, Rotterdam and Antwerp soared to 408 dollars^55^. After some abnormal countries reduced the coal supply to other countries by 80%, Iceland, Ireland, Turkey, Finland, Serbia and Portugal fell into an abnormal state due to the indirect shock. Although these countries have no direct trade relationship with the Russian Federation, they were affected by the indirect shock.
3. Multiple crises in a country also make a country face an abnormal state. After the direct shock caused by the Russian Federation and the shock of the Russian Federation’s second level of propagation through Poland, the Czech Republic fell into an abnormal state. Bulgaria did not import coal directly from the Russian Federation, but it was shocked by the second level of spread caused by the Russian Federation through Romania and Slovenia. The shock that began with the Russian Federation spread to Turkey through Denmark, the Netherlands or Poland and influenced Bulgaria through Turkey.

### Future outlook

During the research process, we made some modifications to the cascading failure model to better align it with real-world scenarios. However, our study still has some limitations. Firstly, in determining whether a country transitions from a normal to an abnormal state, we used the relationship between coal supply and demand, with coal demand data based on the year 2021. However, when coal supply risks occur, countries will inevitably readjust their energy structures to cope with the risks, leading to changes in coal demand. Secondly, concerning the parameter *β*, which represents the proportion of coal exports retained by each country to resist risks, its setting varies among countries in reality.

For addressing the first concern, we are considering whether the coal consumption data for 2022 can be used to represent the coal demand. Currently, we have obtained coal consumption data for some countries from the bp Statistical Review of World Energy published in 2023. However, there are still some countries’ coal consumption data we have not yet found, and we will continue our efforts to locate this information in the future. Regarding the second question, we have not yet identified appropriate indicators to characterize the *β* value for each country. In our future research, we will delve into relevant literature to explore suitable methodologies and solutions to address this issue.

## Data Availability

Data can be obtained from the U.N. Comtrade Database (https://comtradeplus.un.org/), the 71st edition of the bp Statistical Review of World Energy(https://www.bp.com.cn/zh_cn/china/home/news/reports.html), and the International Energy Agency(https://www.iea.org/data-and-statistics/data-product/quarterly-coal-statistics).
